# Brain MRI dataset of multiple sclerosis with consensus manual lesion segmentation and patient meta information

**DOI:** 10.1016/j.dib.2022.108139

**Published:** 2022-04-07

**Authors:** Ali M. Muslim, Syamsiah Mashohor, Gheyath Al Gawwam, Rozi Mahmud, Marsyita binti Hanafi, Osama Alnuaimi, Raad Josephine, Abdullah Dhaifallah Almutairi

**Affiliations:** a Department of Computer Science, Dijlah University College, Baghdad, Iraq; b Department of Computer and Communication System Engineering, Universiti Putra Malaysia, Serdang, Malaysia; c Department of Neurology, Baghdad University, Baghdad, Iraq; d Department of Imaging, Universiti Putra Malaysia, Serdang, Malaysia; e Department of Radiology and Medical Imaging, Elias Emergency University Hospital, Bucharest, Romania

**Keywords:** Expanded disability status scale (EDSS), Automated MS-lesion segmentation, Lesion masks, Gold standard, Ground truth data, T1-weighted, T2-weighted and fluid-attenuated inversion recovery (FLAIR)

## Abstract

Magnetic resonance imaging (MRI) provides a significant key to diagnose and monitor the progression of multiple sclerosis (MS) disease. Manual MS-lesion segmentation, expanded disability status scale (EDSS) and patient's meta information can provide a gold standard for research in terms of automated MS-lesion quantification, automated EDSS prediction and identification of the correlation between MS-lesion and patient disability. In this dataset, we provide a novel multi-sequence MRI dataset of 60 MS patients with consensus manual lesion segmentation, EDSS, general patient information and clinical information. On this dataset, three radiologists and neurologist experts segmented and validated the manual MS-lesion segmentation for three MRI sequences T1-weighted, T2-weighted and fluid-attenuated inversion recovery (FLAIR). The dataset can be used to study the relationship between MS-lesion, EDSS and patient clinical information. Furthermore, it also can be used for the development of automated MS-lesion segmentation, patient disability prediction using MRI and correlation analysis between patient disability and MRI brain abnormalities include MS lesion location, size, number and type.

## Specifications Table

**Table:** 

Subject	Biomedical Engineering
Specific subject area	Medical Image processing, automated lesion segmentation and EDSS prediction, neuroimaging
Type of data	NIfTI Image Table
How the data were acquired	The patient's MRI were acquired on 1.5 Tesla came from twenty different centres with different MRI sequence parameters as listed in supplementary Table 2 while patient's meta information which include general patient information and clinical information was collected from patient files and patient follow-up documents at MS-Clinic.
Data format	Raw and processed
Description of data collection	The data were collected from patients at MS-Clinic if they have confirmed MS disease and complete patient's meta information.
Data source location	• *Institution:* Baghdad Teaching Hospital • *City/Town/Region:* Baghdad /Medical City Complex • *Country:* Iraq
Data accessibility	Repository name: Mendeley Data Data identification number: Direct URL to data https://data.mendeley.com/datasets/8bctsm8jz7/1 OR doi:10.17632/8bctsm8jz7.1

## Value of the Data

• This dataset currently has the largest publicly available number of MS patient's dataset with manual lesion segmentation.
• This dataset currently has the largest general and clinical patient information publicly available.
• This dataset has manual lesion segmentation for three MRI sequences T1-weighted, T2-weighted and fluid-attenuated inversion recovery (FLAIR) done and validated by three experts.
• These data can be employed by biomedical and computer science researchers to detect and quantify MS lesion. Furthermore, it also can be used by neuroscience researchers for evaluating the influence of brain abnormalities on patient disabilities.
• This dataset can be used in different research areas such as automated MS-lesion segmentation, patient disability prediction using MRI and correlation analysis between patient disability and MRI brain abnormalities include MS lesion location, size, number and type.

## 1 Data Description

This dataset includes a 2D FLAIR MRI for 60 patients were collected from patients at MS-Clinic, Baghdad Teaching Hospital, Medical City Complex, Baghdad, Iraq. It consists of 46 females and 14 males with an average age of 33 years ranged from 15 to 56 years, the MRI acquisition dates are between 2019 and 2020, 1.5 Tesla came from 20 centres, average EDSS score [1] of 2.3 ranged between 0 and 6, 78% of patients have EDSS score less than 4 while the rest have EDSS score equal or greater than four. The majority of patients came from Baghdad city while the minority came from all over Iraq. All patients in this dataset have confirmed MS disease by neurologist at MS-Clinic.

The complete patient meta information described in supplementary Table 1 and is publicly available on Mendeley Data repository. The patient meta information includes general patient information such as age, gender, co-morbidity, age of onset, presenting symptom, type of medicines and clinical patient information include the information of patient has an abnormality in any part of neurological examinations. The clinical information also has available neurological examinations which are pyramidal cerebella, brain stem, sensory, sphincters, visual, mental, speech, motor system, sensory system, coordination, gait, bowel and bladder function, mobility, mental state, optic discs, fields, nystagmus, ocular movement, swallowing and EDSS. The difference between the data of MRI acquisition and the date of EDSS scoring is less than two months for 26 patients while the data difference for the rest of patients are greater than two months, two month duration is the regular time for most of MS-patient to complete the diagnosis phase.

The repository has 60 directories for each patient and an excel file contained supplementary Table 1. Each directory contains six files representing three MRI sequences, T1-w, T2-w and FLAIR, and their corresponding consensus manual lesion segmentations providing the lesion masks in the NIfTI format (i.e., files are saved as “file.nii”). NIfTI is one of the most common type of file format for neuroimaging. Table 1 provides a description for each file in the patient directory.

**Table 1:** Illustrate a description for each file in the patient directory.

**No**	**Filename**	**Description**
1	XX-T1.nii	T1 MRI sequence for a patient ID XX in a format of NII
2	XX-T2.nii	T2 MRI sequence for a patient ID XX in a format of NII
3	XX-FLAIR.nii	FLAIR MRI sequence for a patient ID XX in a format of NII
4	XX-LesionSeg-T1.nii	Consensus manual lesion segmentation for T1 MRI sequence for a patient ID XX in a format of NII
5	XX-LesionSeg-T2.nii	Consensus manual lesion segmentation for T2 MRI sequence for a patient ID XX in a format of NII
6	XX-LesionSeg-FLAIR.nii	Consensus manual lesion segmentation for FLAIR MRI sequence for a patient ID XX in a format of NII

## 2 Experimental Design, Materials and Methods

This dataset includes raw images, EDSS, general patient information, clinical information and consensus manual lesion segmentation done by three experts (two radiologists and one neurologist). The dataset was collected from patients with confirm MS disease at MS-Clinic between 2019 and 2020. The inclusion criteria for the collected patients are: First, the patient must have FLAIR, T1-w and T2-w MRI sequences. Second, the MRI scan is using 1.5 Tesla. Third, the patient must have EDSS, general patient information and clinical patient information.

**Fig. 1:**
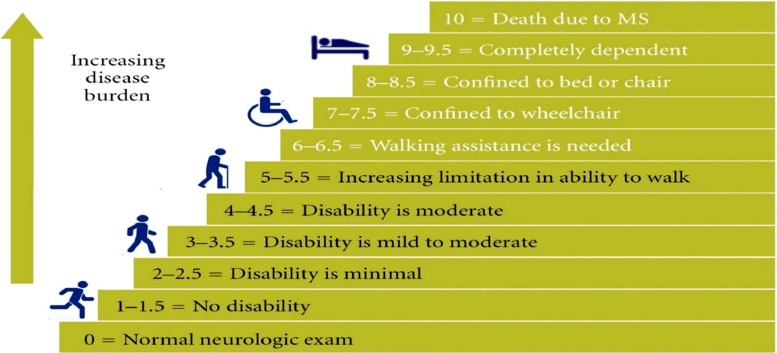
EDSS scores range with its corresponding disability stage as well as the progression of the disease [1].

EDSS was scored by a neurologist at MS-Clinic and EDSS is used to score the level of MS clinical patient's disability [1]. EDSS is a clinician-administered assessment scale used to evaluate the functional systems of the central nervous system [2]. EDSS scores range between 0 (no disability) to 10 (death due to MS) with an increment interval of 0.5. Fig. 1 shows EDSS scores range with its corresponding disability stage as well as the progression of the disease. EDSS scores for the majority of MS-patients are ranged from 0 to 4.5. To assist EDSS, eight neurological Functional System (FS) examinations should be scored by an expert. These eight FSs in total, together with ambulation are visual, brainstem, pyramidal, cerebellar, sensory, bowel and bladder, cerebral and ambulation [3], each one controls specific function of human body. The scoring range for these eight neurological FS examinations are between 0 and 4 to 0–15 [1], the lowest score means normal FS while the highest score means complete loss of function in a particular neurological FS.

Segmentation and validation of the manual lesion segmentation are done by three experts, two radiologists and one neurologist in slice-by-slice manner for three MRI sequences T1-w, T2-w and FLAIR. The whole process can be summarized by three steps. The first step was done by a radiologist resident with three years of experience to perform slice-by-slice for all patients. The second step was done by a consultant radiologist with ten years of experience to validate and correct the incorrect manual lesion segmentation done in the first step. The third step was done by a consultant neurologist with eleven years of experience at MS-clinic to validate and correct the incorrect manual lesion segmentation done in the second step. Three layers of segmentation and validation steps are aimed to ensure the accuracy of the segmented regions and to support the correct diagnosis with aid of meta data.

## Ethics Statements

The data collection was approved by the ethics committee of MS-clinic, Baghdad Teaching Hospital, Medical City Complex, Baghdad, Iraq and written approval was obtained from all patients.

## CRediT authorship contribution statement

**Ali M. Muslim:** Investigation, Resources, Data curation, Writing – review & editing, Writing – original draft, Methodology, Software. **Syamsiah Mashohor:** Supervision, Formal analysis, Writing – review & editing, Writing – original draft. **Gheyath Al Gawwam:** Supervision, Validation. **Rozi Mahmud:** Supervision, Methodology. **Marsyita binti Hanafi:** Supervision, Methodology. **Osama Alnuaimi:** Validation, Methodology. **Raad Josephine:** Validation, Methodology. **Abdullah Dhaifallah Almutairi:** Validation, Methodology.

## Declaration of Competing Interest

The authors declare that they have no known competing financial interests or personal relationships that could have appeared to influence the work reported in this paper.

## Data Availability

Brain MRI Dataset of Multiple Sclerosis with Consensus Manual Lesion Segmentation and Patient Meta Information (Original data) (Mendeley Data).

## References

[1] Kurtzke J.F. (1983). Rating neurologic impairment in multiple sclerosis: an expanded disability status scale (EDSS). Neurology.

[2] Meyer-Moock S., Feng Y.S., Maeurer M., Dippel F.W., Kohlmann T. (2014). Systematic literature review and validity evaluation of the expanded disability status scale (EDSS) and the multiple sclerosis functional composite (MSFC) in patients with multiple sclerosis. BMC Neurol.

[3] Şen S. (2018). Neurostatus and EDSS calculation with cases. Noro Psikiyatr. Ars..

